# Effect of 3D-scaffold formation on differentiation and survival in human neural progenitor cells

**DOI:** 10.1186/1475-925X-9-70

**Published:** 2010-11-11

**Authors:** Stefanie Ortinau, Jürgen Schmich, Stephan Block, Andrea Liedmann, Ludwig Jonas, Dieter G Weiss, Christiane A Helm, Arndt Rolfs, Moritz J Frech

**Affiliations:** 1 Albrecht-Kossel-Institute for Neuroregeneration, University of Rostock, Gehlsheimerstrasse 20, 18147 Rostock, Germany; 2 Institute for Physics, Ernst-Moritz-Arndt University Greifswald, Felix-Hausdorff-Str. 6, 17489 Greifswald, Germany; 3 Electron Microscopic Centre, Institute of Pathology, University of Rostock, Strempelstrasse 14, 18055 Rostock, Germany; 4 Division of Cell Biology and Biosystems Technology, Institute of Biological Sciences, University of Rostock, Albert-Einstein-Strasse 3, 18051 Rostock, Germany

## Abstract

**Background:**

3D-scaffolds have been shown to direct cell growth and differentiation in many different cell types, with the formation and functionalisation of the 3D-microenvironment being important in determining the fate of the embedded cells. Here we used a hydrogel-based scaffold to investigate the influences of matrix concentration and functionalisation with laminin on the formation of the scaffolds, and the effect of these scaffolds on human neural progenitor cells cultured within them.

**Methods:**

In this study we used different concentrations of the hydrogel-based matrix PuraMatrix. In some experiments we functionalised the matrix with laminin I. The impact of concentration and treatment with laminin on the formation of the scaffold was examined with atomic force microscopy. Cells from a human fetal neural progenitor cell line were cultured in the different matrices, as well as in a 2D culture system, and were subsequently analysed with antibody stainings against neuronal markers. In parallel, the survival rate of the cells was determined by a live/dead assay.

**Results:**

Atomic force microscopy measurements demonstrated that the matrices are formed by networks of isolated PuraMatrix fibres and aggregates of fibres. An increase of the hydrogel concentration led to a decrease in the mesh size of the scaffolds and functionalisation with laminin promoted aggregation of the fibres (bundle formation), which further reduces the density of isolated fibres. We showed that laminin-functionalisation is essential for human neural progenitor cells to build up 3D-growth patterns, and that proliferation of the cells is also affected by the concentration of matrix. In addition we found that 3D-cultures enhanced neuronal differentiation and the survival rate of the cells compared to 2D-cultures.

**Conclusions:**

Taken together, we have demonstrated a direct influence of the 3D-scaffold formation on the survival and neuronal differentiation of human neural progenitor cells. These findings emphasize the importance of optimizing 3D-scaffolds protocols prior to *in vivo *engraftment of stem and progenitor cells in the context of regenerative medicine.

## Background

Tissue engineering is an interdisciplinary field combining biological sciences and engineering to develop tissues that restore, maintain or enhance tissue function. In the context of regenerative medicine, the combination of biomaterial scaffolds with neural stem and progenitor cells holds great promise as a therapeutic tool [1,2]. 3D-matrices have been generated from various materials such as poly L-lactic acid and poly glycolic acid [3,4], as well as biopolymers such as collagen, fibrin, and alginate [5-14]. The hydrogel-based PuraMatrix is an ionic self-complementary amphiphilic oligopeptide hydrogel matrix, able to form 3D nano-scaffolds consisting of β-sheets and fibres by spontaneous molecular self-assembling [12-14]. The hydrogel scaffold is widely used in the areas of tissue engineering and stem cell research, and it has been shown to promote differentiation in different cell types [15-24]. As the composition, concentration and functionalisation of the 3D-scaffolds is essential for cell adhesion, growth and differentiation, we varied the concentration and functionalisation of PuraMatrix seeded with a human neural progenitor cell line (ReNcell VM, Millipore, USA) and studied the effects on the assembly of the matrix, and subsequently the influence on the differentiation of the human progenitor cells. This cell line shows fast proliferation and can be cultured easily, which makes it an appropriate system to test the influence of a 3D environment. The cells can be differentiated into neurons, astrocytes and oligodendrocytes within a few days by simple withdrawal of growth factors [25]. Although the overall differentiation into neuronal cells is relatively low, the above mentioned properties made the cell line an appropriate model to study differentiation of human progenitor cells, and has already been used in other studies [26-29]. Matrix assembly was analysed by performing atomic force microscopy to provide structural information about the matrix, such as spatial dimensions of the fibres and the structure of the network formed by these fibres. In addition we report data on the influence of the scaffold formation and functionalisation on proliferation, growth and differentiation of human neural progenitor cells cultured in the 3D-scaffolds in comparison to the situation in 2D cultures. The data presented provides new information for optimizing 3D-scaffolds to be used in die field of regenerative medicine.

## Methods

### Atomic force microscope (AFM) measurements

PuraMatrix (PM; BD Biosciences, Heidelberg, Germany) and laminin stock solutions were kept at 4°C until used. Solutions for the deposition process were freshly prepared from the stock solutions as described below, but without cells. Muscovite mica sheets were freshly cleaved and immediately placed into a 24 well-plate. In each case 100 μl of the solutions were placed on top of the mica sheet, then 400 μl of media without growth factors was added. One day later the mica sheets were rinsed in ultra pure water (Millipore, Billerica, MA) to remove excess gel loosely bound to the mica, and then dried under a stream of nitrogen.

Imaging was performed using a Multimode Atomic Force Microscope (AFM) with Nanoscope IIIa controller and "E" scanner (Digital Instruments, Santa Barbara, CA). The "E" scanner exhibits a maximum scan area of 10 × 10 μm^2^, a vertical range of 2.5 μm and was height calibrated using a TGZ01 grating (MicroMasch, Estonia; step height 26 nm) and linearised using a PG grating (Digital Instruments; 1 μm pitch). The images were recorded with tapping mode in air using standard tapping mode cantilevers (OMCL-AC160TS, Olympus). Imaging in fluid was not necessary because the drying process does not affect the structure of the scaffold [30].

Before usage the cantilevers were tested with a Nioprobe self-imaging sample (Aurora Nanodevices, Canada) and with a gold cluster sample (cluster radius < 15 nm; synthesised according to [31]) and only cantilevers with tip radius *R *< 10 nm were used for imaging. Images were obtained from at least five different positions and in three different resolutions (500 nm × 500 nm, 1500 nm × 1500 nm, 10 μm × 10 μm).

Image processing was performed using homebuilt scripts in MatLab (MathWorks Natick, MA). The large area scans (1500 nm × 1500 nm and 10 μm × 10 μm) were used to get an impression of the coarse network structure (i.e. number of crossing points, aggregation of single fibres etc.) whereas the small area scans (500 nm × 500 nm) were used to measure the geometric properties of PM fibres in presence and absence of laminin.

**Figure 1:**
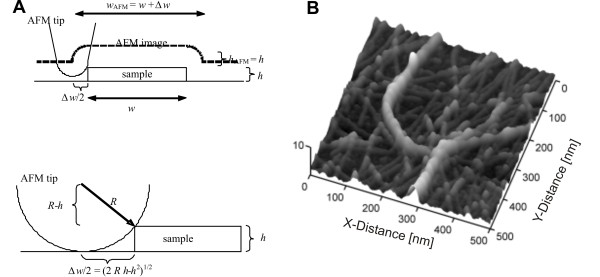
**Interaction of the AFM tip with a PM fibre**. (A) Interaction of the AFM tip with a PuraMatrix fibre can be well approximated by the interaction of a sphere (radius *R*) with a cuboid (width *w *and height *h*). The upper scheme shows that the spatial extension of the AFM tip leads to a broadening of the slab width *w*_*AFM *_in the AFM image whereas the cuboid height remains unaffected, i.e. *h*_*AFM *_= *h*. (B) AFM image (PM 0.15% sample) in 3D view with false colour coding: brighter structures are higher than darker ones. AFM provides us with structural information in three dimensions, which allows us determine the structure of the network formed by these fibres.

It is well known that PM fibres form beta-sheets [32] and hence the interaction of the AFM tip with one PM fibre can be well approximated by the interaction of a sphere (radius *R*) with a cuboid (width *w *and height *h*). From Figure 1A one can conclude that the spatial extension of the AFM tip leads to a broadening of the slab width *w*_AFM _in the AFM image whereas the cuboid height *h*_AFM _remains unaffected. Using simple geometric calculations one can estimate that the difference between measured and true width is given by (Figure 1A bottom).

(1) $$w_{\text{AFM}}-w=2{\left(2\;h\;R-h^{2}\right)}^{1/2}.$$

This introduces a noticeable error in our case as the dimensions of tip radius (approx. 5 nm) and PM fibre height (approx. 1.3 nm) and width (approx. 5 nm) are of the same order of magnitude. To account for this effect the curvature radius of the AFM tip *R *was obtained from tapping mode images of the Nioprobe self-imaging sample. Furthermore, the width *w *and the height *h *of isolated PM fibres were measured from 500 nm × 500 nm scans of PM samples (PM concentration 0.15%, with and without laminin) and corrected according to Eq. 1. The error given in the results was calculated using the standard deviation (tip radius *R *and beta-sheet height *h*) or by employing the error propagation law (beta-sheet width *w*).

### Cell culture

2D culture of ReNcell VM (Millipore, Schwalbach, Germany) cells was carried out as described previously [27]. Cells were cultivated on laminin I (mouse laminin I, AMS Biotechnology, Germany) coated flasks or chamber slides in Dulbecco's modified eagle medium (DMEM)/F12, supplemented with Glutamax, B27 media supplement, heparin sodium salt and gentamycin (all Invitrogen, Karlsruhe, Germany). Epidermal growth factor (20 ng/ml; EGF) and basic fibroblast growth factor (10 ng/ml; bFGF; both Roche, Mannheim, Germany) were added to the media during proliferation. Differentiation of the cells was induced by withdrawal of the EGF and bFGF.

To prepare 3D PuraMatrix hydrogel matrices (BD Biosciences, Heidelberg, Germany) 2D cultured cells were trypsinized and resuspended in 10% sucrose (60,000 cells/100 μl matrix) and incubated for 30 min. For functionalisation of the PuraMatrix, cells were mixed with laminin I solution (mouse laminin I, AMS Biotechnology, Germany) in advance to the incubation (8 μg/100 μl matrix). Subsequently cells were transferred to coverslips in a 24-well plate and media (400 μl/well) was added. Afterwards the matrices were allowed to gelate for 1 h. Matrices were washed with 500 μl media for 10 min at room temperature. After an additional washing step the matrices were incubated at 37°C/5% CO_2_. Unless otherwise stated all reagents were purchased from Sigma (Taufkirchen, Germany).

### Immunocytochemistry and scanning electron microscopy

For immunocytochemistry, 2D- and 3D-cultures were fixed with paraformaldehyde (4% in 0.1 M PBS) for 20 min. Cells were incubated with the primary antibody over night at 4°C (anti-β-III-tubulin, 1:1000, mouse, Sigma, Munich, Germany) or anti-tyrosine hydroxylase (TH, 1:500, mouse, R & D Systems, Wiesbaden, Germany). Secondary antibody (1:1000; goat, anti-mouse Alexa Fluor 488, Molecular Probes) was added for 5h at room temperature. Cell nuclei labelling was performed with 4',6-Diamidin-2'-phenylindoldihydrochlorid (DAPI, 100 ng/ml in PBS, Sigma, Munich, Germany). 3D-matrices and 2D-cultures were analysed either by fluorescent microscopy (Olympus BX51, Olympus Germany or a Biozero microscope, Keyence, Germany, Karlsruhe), or confocal microscopy (Leica, DM IRE2 equipped with UV, Argon and Xenon lasers).

In 2D-cultures, pictures were taken of 8-10 non overlapping visual areas per chamber, with three chambers per time point. DAPI stained nuclei were counted using GSA Image Analyzer program (GSA, Rostock, Germany), whereas immuno-positive cells were counted manually.

For 3D-matrix cultures, at least three stacks of images (20-40 images/stack, distance 2 μm) per time point and per concentration of PM sample were taken by confocal microscopy. To obtain the total cell numbers, stacks were merged with the NIS element D program (Nikon, Düsseldorf, Germany) and then analysed with GSA Image Analyzer program. Given values represent mean percentage ± SEM with respect to DAPI stained cells. Data were obtained from at least 3 independent experiments (N) and ≥6 measurements (n) per N for 2D cultures and ≥3 (n) for 3D-experiments.

For scanning electron microscopy, cells contained in PuraMatrix were fixed with glutaraldehyde (4% in PBS) for 1 h or overnight, rinsed with PBS and subsequently dehydrated in acetone with increasing concentrations (30%, 50%, 75%, 90%, 100%). Specimens were dried with a critical point drier (BalTec, Germany) and sputter coated with gold. Pictures were taken with a DSM 960A scanning electron microscope (ZEISS, Germany).

### Live/dead assay

Viability of cells was measured by live/dead assay (Molecular Probes, Karlsruhe, Germany) and analysed by fluorescence microscopy (TS100, Nikon, Düsseldorf, Germany). Quantitative data of 2D-cultures were obtained by manually counting of 8-10 non-overlapping areas per three chambers per time point. 3D-matrices were analysed by taking at least three image stacks per matrix concentration and time point. Viability is given as mean ± SEM of living cells in the total number of cells.

### Statistics

All statistical analyses described were performed with Prism 5 (GraphPad Prism. Inc., USA) using one way ANOVA analysis with the Bonferroni Post test. p-value ≤ 0.05 (indicated by *) was considered to indicate significant statistical differences. Values represent mean ± sem.

## Results

### Atomic force microscopy of PuraMatrix

**Figure 2:**
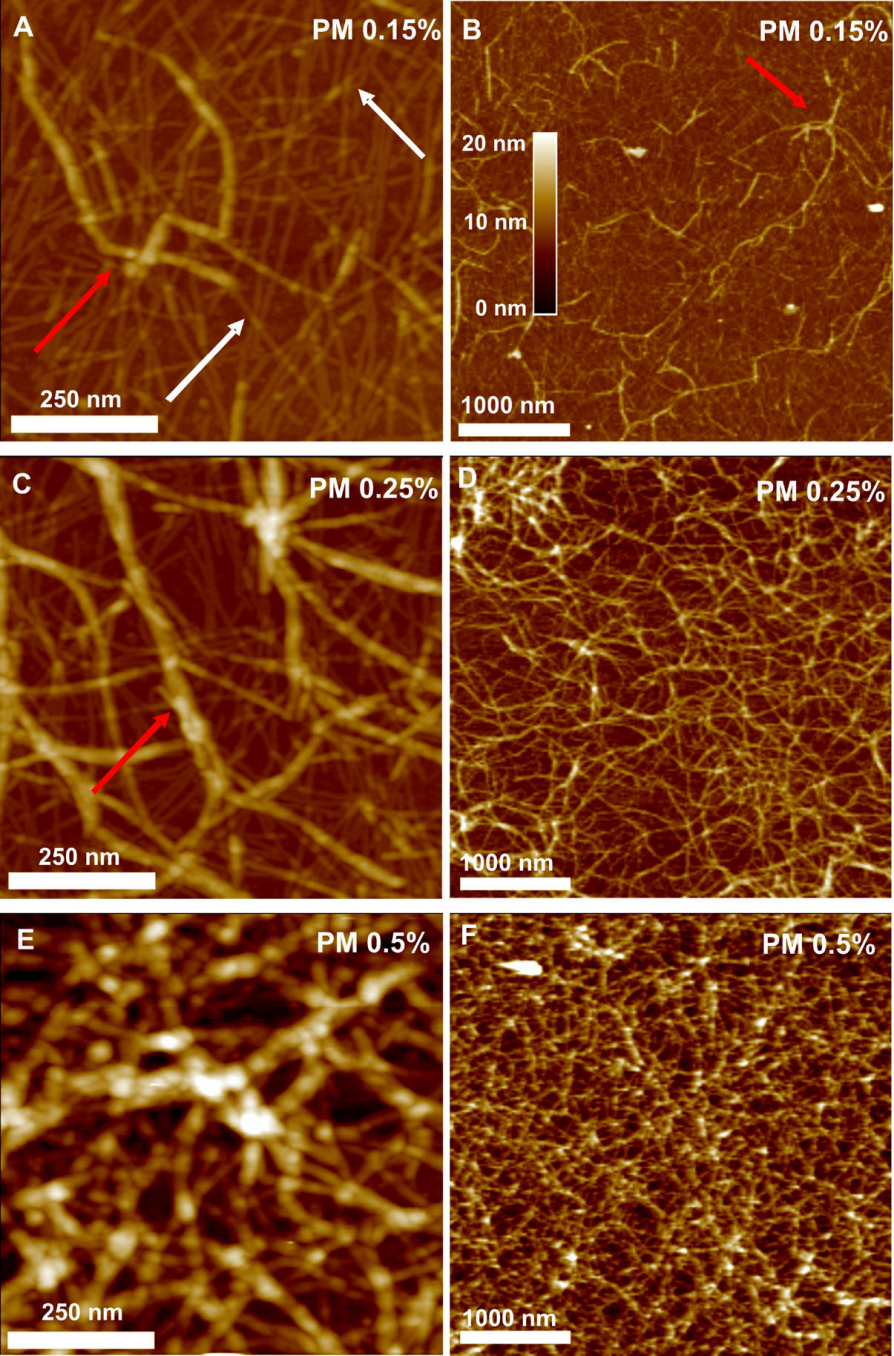
**AFM images of pure PuraMatrix scaffolds**. AFM images of pure PuraMatrix scaffolds; scan size: 0.75 × 0.75 μm^2 ^(left column) or 4 × 4 μm^2 ^(right column). Depending on the PM concentration two kinds of fibres are formed: long and thin beta-sheets (i.e. isolated PM fibres, exemplary marked by white arrows; height ca. 1.3 nm and length > 1 μm) and bundles or aggregates of these sheets (red arrows). Additionally, a crossing of single PM fibres is accompanied by an increase in height (white arrows), indicating that once the PM fibres are created, they do not interdigitate. At low PM concentrations (PM 0.15% and 0.25%, Fig. 2A-D) the samples show a homogeneous distribution of single PM fibres and bundles, whereas an increase in PM concentration rises the numbers of bundles within the network. At 0.5% PM concentration (E, F) the fibres form very dense networks that cannot be penetrated by AFM.

**Figure 3:**
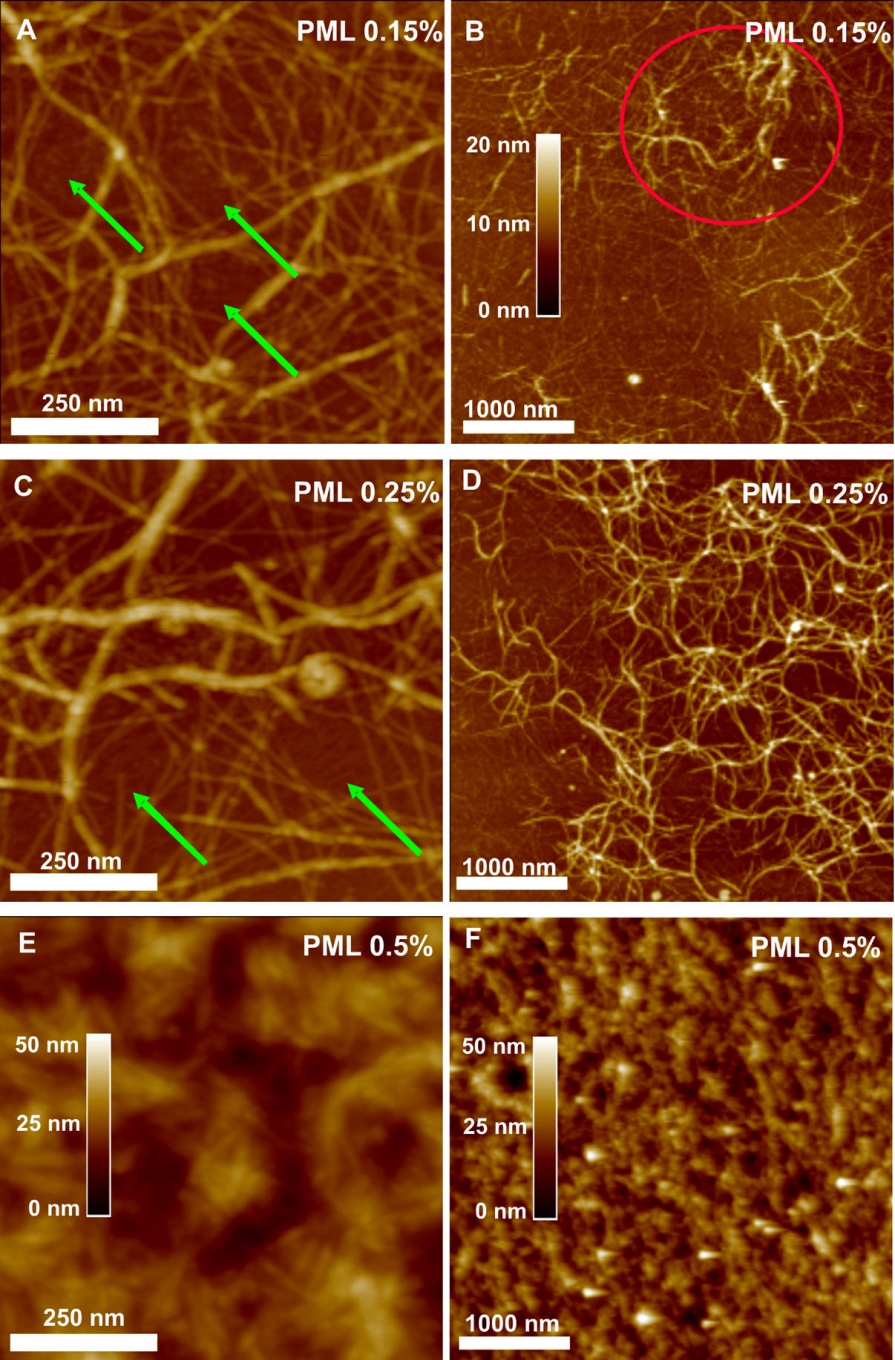
**AFM images of PuraMatrix scaffolds after addition of laminin I**. AFM images of PuraMatrix + laminin I scaffolds; scan size: 0.75 × 0.75 μm^2 ^(left column) or 4 × 4 μm^2 ^(right column). Again, bundles and single PM fibres are observed. In contrast to pure PM scaffolds, bundles are frequent even at 0.15% PM concentration and inhomogeneously distributed over the sample (red circle in Fig. 3B). Between these coarse bundle-networks single PM fibres can be found that are less densely distributed than in the non-functionalised scaffold (green arrows, indicating a reduced surface coverage of PM fibres in comparison to Fig. 2A, C). Hence, the addition of laminin I promotes bundling and decreases the density of isolated PM fibres.

The composition, concentration and functionalisation of a 3D-scaffold is essential for cell adhesion, growth and finally differentiation. Therefore we studied matrix assembly by performing atomic force microscopy (AFM) on PuraMatrix samples. AFM images provide structural information in three dimensions which allowed us to measure the spatial dimensions of the fibres as well as the structure of the network formed by these fibres (Figure 1B). For pure PM scaffolds the AFM images are given in Figure 2, whereas Figure 3 shows the AFM results for scaffolds made by a mixture of PM and laminin I. In these figures the surface morphology is given in two different resolutions: the left column shows the scaffold morphology at a surface area of 0.75 × 0.75 μm^2^, which allows the resolution of single, isolated PM fibres, whereas the right column has a surface area of 4 × 4 μm^2 ^and is used to study the structure of the scaffold on a much larger scale. For pure PM scaffolds made at low PM concentration (Figure 2A, B; PM 0.15%) most of the scaffold consists of thin PM fibres (e.g. white arrows in Figure 2A) which are quite homogeneously distributed over the scaffold and mostly isolated. Interestingly, these fibres show an alignment which is parallel to the mica surface. Therefore we conclude that the washing of the PM samples by Milli-Q water leads to a dissection of the PM scaffold and that only the fibres and aggregates of the scaffold, which are adjacent to the mica surface, remain on the sample. Due to this alignment we were able to measure the geometric properties of the pure and isolated PM fibres and found that they have an average height of 1.28 ± 0.16 nm and an average width of 6.86 ± 2.69 nm, which agrees well with the molecular dimensions of single PuraMatrix beta-sheets (height: 1.3 nm, width: 5 nm, according to [30]). However, we also found structures in the AFM images whose geometric properties are larger (in height and width) than the isolated PM fibres (red arrows in Figure 2A, B). We attribute these structures to aggregation of single PM fibres into PM bundles. Interestingly, the amount of bundles rises with increasing PM concentration (Figure 2C - F), which causes very dense scaffolds at high PM concentrations (Figure 2E, F). The higher resolved AFM images show (left column of Figure 2) that for pure PM scaffolds there is always a homogeneous distribution of single PM fibres within the scaffold. An increase in PM concentration leads to increased aggregation (bundle formation) of PM fibres. On the other hand, it can be seen from the larger AFM scans (right column of Figure 2), that the PM bundles form a coarser network themselves. The PM bundles of this network are also quite homogeneously distributed over the surface, whereas the mesh size increases with decreasing PM concentration (Figure 2D, F). At 0.15% PM concentration bundle formation becomes infrequent and we find almost no PM bundle network within the scaffold (Figure 2B). This is changed upon addition of laminin (red circle in Figure 3B). Here the scaffold becomes dense only at 0.5% PM concentration (Figure 3E, F). At lower concentrations single PM fibres become less frequent and most of the network is formed by PM bundles (Figure 3A-D). Additionally, these bundle networks are inhomogeneously distributed over the surface (Figure 3B, D). Hence, we conclude that the addition of laminin promotes bundle formation and reduces the amount of isolated PM fibres. As the same PM amount is now distributed more in bundles and less in single sheets, we observed areas surrounded by the bundle network which are not covered by any PM fibres (green arrows in Figure 3A, C). This shows that at low PM concentrations the scaffold becomes less dense compared to pure PM scaffolds and hence, that the addition of laminin I has a big impact on the PM scaffold structure.

**Figure 4:**
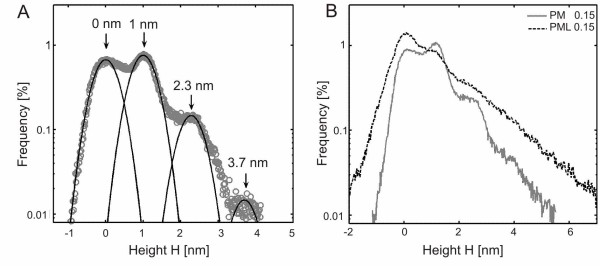
**Height distribution histograms**. (A) Height distribution histograms (open circles, grey) of a PM 0.15% sample and fits to Gaussian distribution function (solid lines). The leftmost peak corresponds to the surface bearing the PM network and was set to 0 nm. Three peaks with an average separation of 1.23 ± 0.21 nm and declining intensities are shown. Therefore mostly single PM fibres are formed and the formation of vertical aggregates is very unlikely. (B) Comparison of height distribution histograms from PM 0.15% (gray line) and PML 0.15% (black line). Obviously the formation of single PM fibres is reduced (peak at approx. 1 nm) and the aggregation to bundles is preferred if laminin is added. Furthermore the surface peak (0 nm) is increased, indicating that less surface is covered by PM. This supports the observation (Fig. 3A, C), that the bundling makes the network less dense.

For a quantification of this effect we calculated height distribution histograms of the AFM images for both types of PM scaffolds at low PM concentrations. Without laminin (PM 0.15% in Figure 4A) a peak can be found around 1 nm in the distribution histogram, corresponding to the height of a single PM fibre (height approx. 1.3 nm) and two peaks around 2.3 nm and 3.7 nm, indicating aggregates of two or three PM fibres respectively. The average separation of the peaks is 1.23 ± 0.21 nm, which is close to the expected value of 1.3 nm (height of one PM fibre). Figure 4A shows that single PM fibres are more frequent than bundles by roughly a factor of 10. Additionally, Figure 4B compares the height distribution histograms of PM 0.15% and PML 0.15%. After addition of laminin the peak at approx. 1 nm is decreased whereas the other peaks are increased by roughly a factor of 2 indicating more bundles are formed by aggregation of PM fibres. Furthermore, the addition of laminin I increases the substrate peak at 0 nm in the histograms. We conclude that the additional aggregation in lateral (x, y-axis) and vertical dimensions (z-axis) after addition of laminin leads to a lowering of surface coverage by PM, which means that more of the bare substrate is visible to the AFM tip and explains the increased substrate peak. Qualitatively, the same result was found in the AFM images (Figure 2A and 3A), where we observed areas without any PM fibres after the addition of laminin (green arrows in Figure 3A). Comparing Figure 4A with Figure 2A, we conclude that peaks at multiples of 1.3 nm are mainly caused by two processes: 1. sandwich-like aggregation of PM fibres to bundles and 2. crossing of two beta-sheets leading to an elevation at the crossover. The second process can be found in Figure 2A, where the crossing points of different PM fibres are marked by white arrows. Interestingly, at every crossing point the height of the fibres locally increases, which indicates that one fibre has to go above the other one and that the fibres do not interdigitate. Furthermore, the PM fibres have to be surprisingly elastic, as every single fibre crossover has similar spatial dimensions as a beta-sheet (same in height but slightly increased width). Hence, the beta-sheets strongly deform just at the crossing point.

### Cell growth in 3D-matrices

As we demonstrated the influence of the PM concentration and of laminin on the formation of the 3D-scaffold, in a subsequent set of experiments we investigated the impact of the scaffold formation on cell growth and survival. We used the human fetal neural progenitor ReNcell VM cell line as a model system for neuronal differentiation as the cells can be differentiated in neuronal cells with a dopaminergic phenotype, and the cells start differentiation within 24 h [26,27].

**Figure 5:**
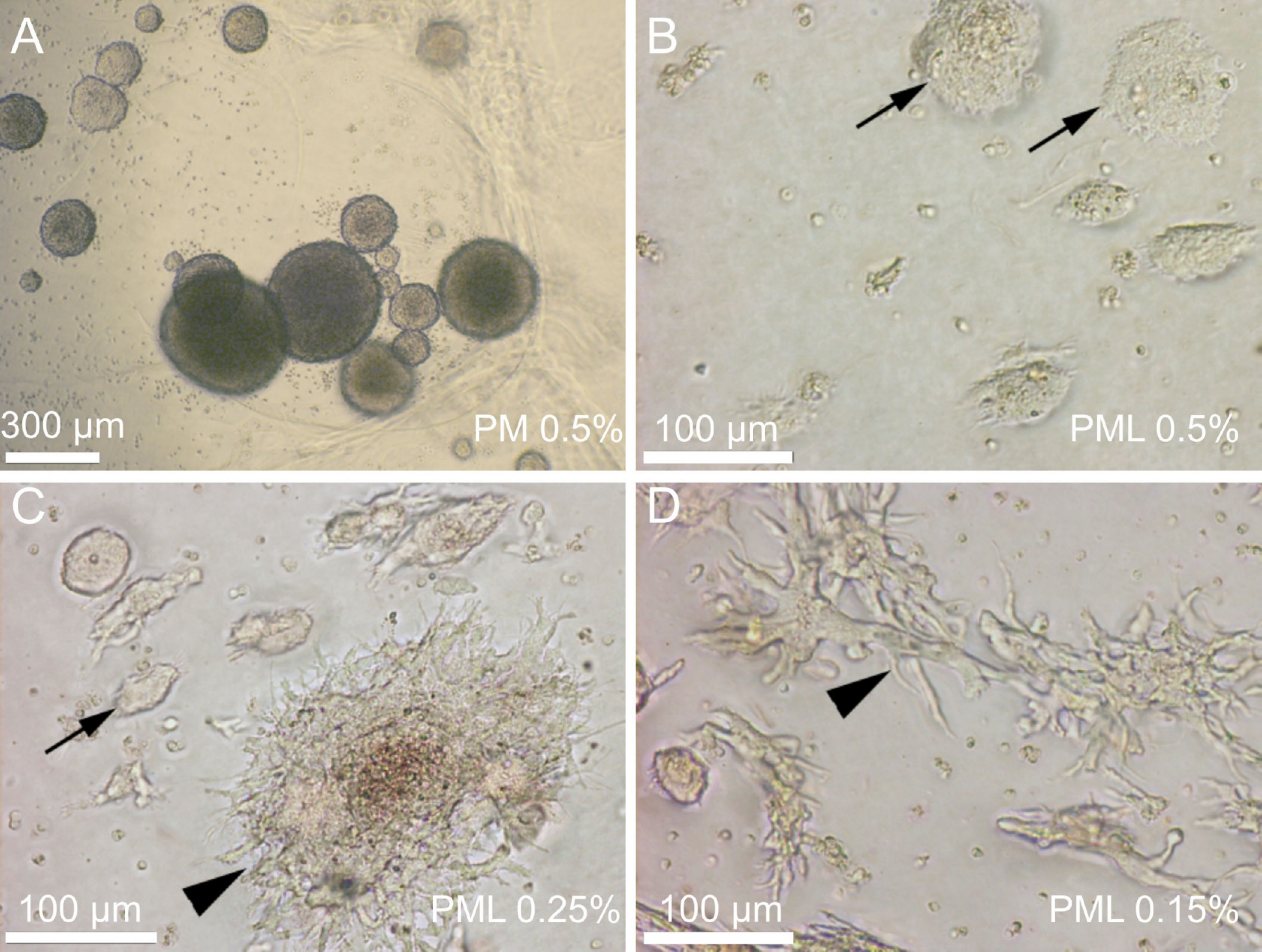
**Growth and proliferation of ReNcell VM cells in 3D PuraMatrix**. (A) 3D culture with 0.5% PM without laminin results in the development of spheroid structures after 5 days of cultivation. (B) Cultivation with 0.5% PM with laminin functionalisation resulted in flat and densely packed cell aggregates. (C) Cultures with 0.25% PM and with laminin functionalisation showed cell aggregates, similar to PML 0.5%, but also loosely composed 3D cellular structures are formed. (D) PML 0.15%, the cellular composition in the matrix is mostly built up of 3D structures.

The cells grown in the hydrogel nanoscaffolds demonstrate a similar proliferating profile to those of 2D cultures with a 24-30 h doubling rate. In all three concentrations a spheroid growth pattern was observed, as shown in Figure 5A. After the functionalisation of the matrix with laminin, the growth pattern of ReNcell VM cells was different. Cells hosted in PuraMatrix/laminin 0.5% scaffolds expressed flat and densely packed cell aggregates (Figure 5B). More loosely composed 3D structures of cells were rarely observed, in those cases mainly after 10 days of cultivation (data not shown). Progenitor cells cultivated in PuraMatrix/laminin 0.25% showed an increased number of 3D-structures which were already developed after 5 days of cultivation, whereas the number of aggregated forms decreased (Figure 5C). Almost all growth patterns in the lowest concentration of PuraMatrix/laminin (0.15%) showed these 3D-structures of ReNcell VM cells. Similar to PuraMatrix/laminin 0.25% 3D structures were already present after 5 days (Figure 5D). Correspondingly, almost none of the cell aggregates were found within these approaches. These results hint at the conclusion that ReNcell VM cells cultured without surface functionalisation grow in neurospheres, whereas in laminin functionalised matrices the growth pattern of the progenitor cells differs in such a way that high concentrations of the scaffold support the growth of cell aggregates whereas decreasing PuraMatrix concentrations lead to extended 3D-growth of ReNcell VM cells.

### Survival of cells in 3D-matrices and 2D-cultivation

**Figure 6:**
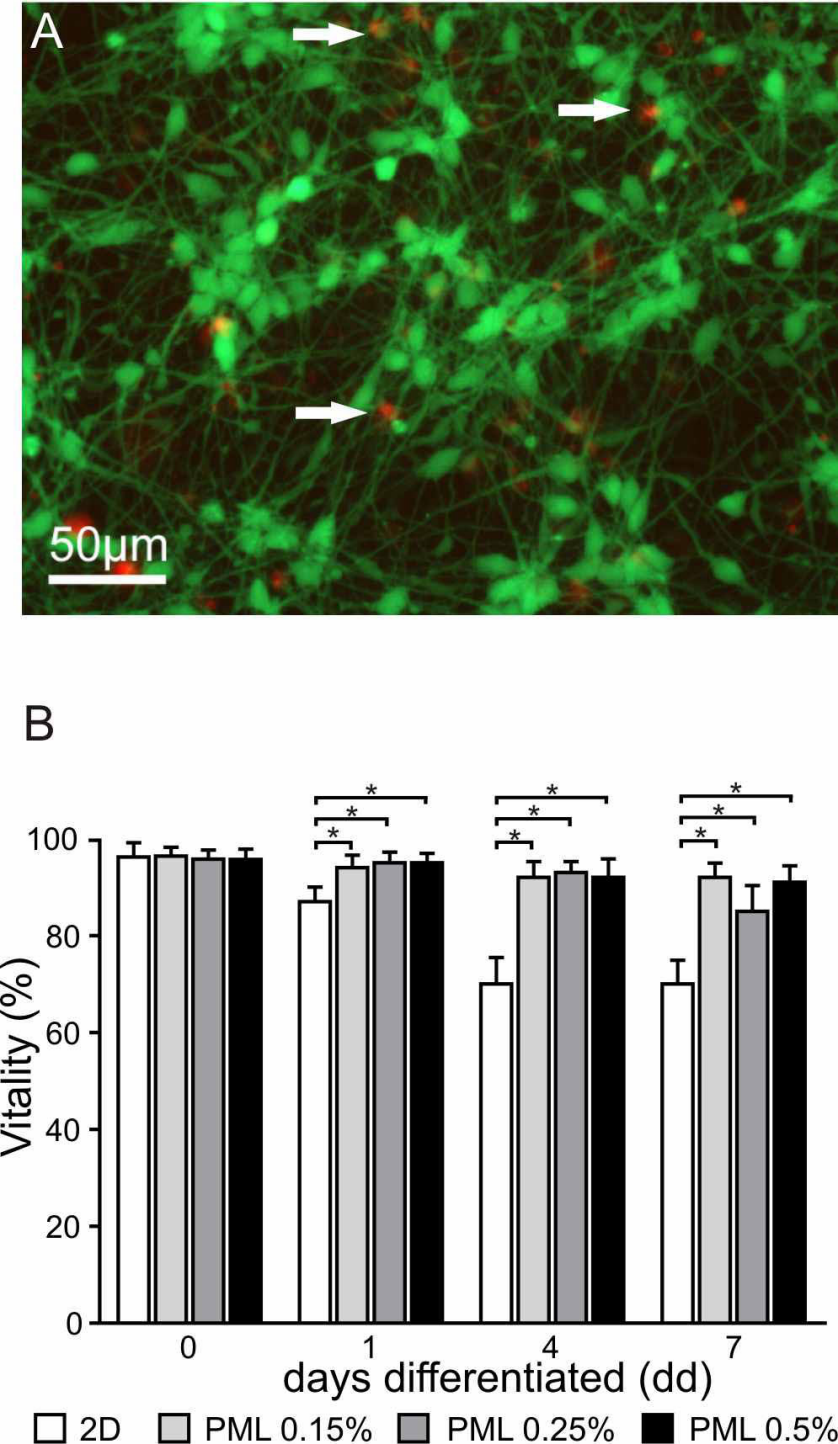
**Live/Dead Assay**. (A) Live/Dead fluorescence image of ReNcell VM cells in 2D-culture conditions. Green fluorescent cells are alive, red fluorescent nuclei indicate dead cells (arrows). (B) Quantification of the stainings by manual counting of living and dead cells of 2D and 3D cultures with different concentrations of PML matrices. Notice the increase of dead cells during differentiation which is significantly higher in the 2D compared to 3D cultures. * indicates p ≤ 0.05.

The survival rate of cells cultured in 3D-scaffolds with different PuraMatrix concentrations was analysed by using a Live/Dead assay, performed at days 0, 1, 4, and 7. To determine if there was a benefit of the 3D-matrix over a 2D-culture system we conducted in parallel a live/dead assay in 2D-cultures. An example picture of a live/dead-assay conducted with cells differentiated for 4 days in a 2D-culture is shown in Figure 6A. Cells differentiated in the 2D-culture system showed a significant increase of dead cells over the time in comparison to day 0 differentiation (Figure 6B, p < 0.005). In comparison no increase of dead cells was observed in the different 3D-scaffolds. Regardless of the PuraMatrix concentration the number of dead cells was stable over the time of differentiation (Figure 6B). The results at day 0 demonstrated in all samples a survival rate higher than 95%. At day 1 the percentage of cell survival in 2D-cultures was about 88% and it decreased further until about 70% at day 7. The lowest survival rate within the three different matrix concentrations was about 85% (PML 0.25%, d7) and therefore only 10% lower compared to the starting point at day 0, although this rate was not significantly different from day 0. From day 1 up to day 7 the survival rates in the 3D-matrices were always significantly higher than in the 2D cultures (p ≤ 0.005), indicating that 3D-matrices support the survival of ReNcell VM cells during differentiation.

### Neuronal differentiation in 3D-matrices and 2D-cultivation

**Figure 7:**
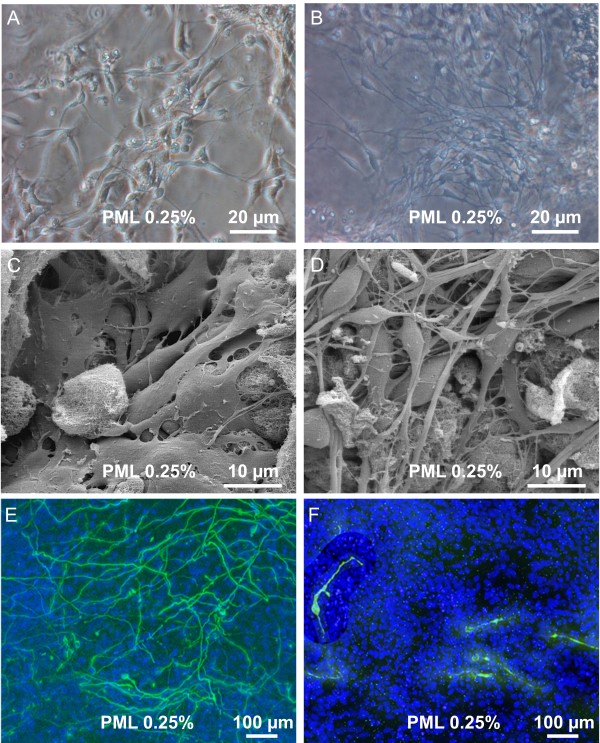
**Proliferation and differentiation in 3D scaffold**. (A + B) Transmission light picture of proliferating cells in PML 0.25% and differentiated cells in PML 0.25%. (C + D) Scanning electron microscope picture of proliferating cells and differentiated cells in PML 0.25%. Upon induction of differentiation one observes the development of a dense 3 dimensional network of processes. (E) Immunocytochemistry for βIII-tubulin and TH (F) of cells in PML 0.25% after 7 days of differentiation revealed a dense network of βIII-tubulin positive cells. TH^+ ^cells were found to possess processes, but without building up a dense network.

Antibody stainings against β-III-tubulin (βIII-tub) and tyrosine hydroxylase (TH) were performed to investigate the influence of the 3D-composition on the number of neurons compared to 2D. The percentage of neuronal cells was determined at day 0, and at day 1, 4 and 7 after differentiation. Cells were proliferated for up to 4 days in scaffolds consisting of different concentrations of PuraMatrix. Figure 7A shows a transmission light picture of proliferating cells (PM 0.25% with laminin). Cell bodies with only a few short processes can be seen, a morphology comparable to 2D cultures (data not shown). Investigation of proliferating cells with a scanning electron microscope revealed cell bodies which are embedded in the PuraMatrix (Figure 7C). Upon induction of differentiation, cells started to develop a dense network of processes (Figure 7B and 7D) and began to the express βIII-tub (Figure 7E) and TH (Figure 7F).

**Figure 8:**
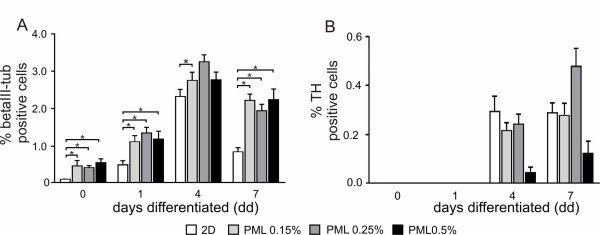
**Quantitative analysis of βIII-tubulin and TH expression**. (A + B) Percentage of βIII-tubulin positive and TH positive cells, respectively, in differentiating ReNcell VM cells grown in 2D and 3D cultures. In all conditions a higher number of βIII-tubulin^+ ^cells was found in the 3D scaffolds in comparison to the 2D cultures. A PuraMatrix concentration of 0.5% was found to be adverse for TH^+ ^cells as the number was lower in these scaffolds, however the difference was not significant. * indicates p ≤ 0.05.

In proliferating cells, the percentage of βIII-tub positive cells (βIII-tub^+^) ranged between 0.4% and 0.5% within the three PuraMatrix/laminin concentrations and was significantly higher in all three PuraMatrix/laminin concentrations compared to the 2D-culture (0.06 ± 0.02%) (Figure 8A). After one day of differentiation the number of neurons had more than doubled within the PuraMatrix/laminin concentrations, compared to 2D-cultures. At day 7 the number of βIII-tub^+ ^cells decreased both within the 3D-scaffolds with all PuraMatrix/laminin concentrations, and in the 2D-cultures. However, in all conditions of the 3D-cultures the number of βIII-tub^+ ^cells was still about 2%, higher in comparison to the 2D-cultures (0.85 ± 0.09%).

An example of the detection of TH positive (TH^+^) cells is shown in Figure 7F. At day 0 and 1 of differentiation in none of the conditions TH^+ ^cells were found. Expression of TH was confirmed at day 4, where 0.2% - 0.3% TH^+ ^cells were detected within the 2D-culture as well as the two lower concentrations of the PuraMatrix/laminin matrix. In contrast, in the scaffold with the highest PuraMatrix/laminin concentration only 0.04 ± 0.02% of TH^+ ^cells were found (Figure 8B). At day 7 the scaffolds with a PuraMatrix/laminin concentration of 0.25% showed the highest number of TH^+ ^cells (0.46 ± 0.05%) which was nearly twice the number of TH^+ ^cells in the scaffolds with a PuraMatrix/laminin concentration of 0.15% (0.27 ± 0.04). Like at day 4 the amount TH^+ ^cells was lowest in the scaffolds with a PuraMatrix/laminin concentration of 0.5% (0.11 ± 0.04%). Regardless of this clear difference the number of TH^+ ^cells was not significantly different in any condition. In general, the formation of a 3D-network of TH^+ ^cells can not be confirmed, but TH^+ ^cells can be found on different levels of z-stacks which ensures a 3D-growth.

## Discussion

Three-dimensional matrices composed of nanofibres and the use of neural progenitor cells are emerging areas in regenerative medicine. Both applications by themselves promise success as therapeutic tools for neurodegenerative diseases [1,2,33]. The combination of both methologies shows synergistic effects and improves the outcome over single applications, as shown in different studies dealing with the culture of cells in 3D scaffolds for example in scaffolds consisting of PuraMatrix [15,22,23]. In our study we investigated the growth and differentiation of a human fetal neural progenitor cell line (ReNcell VM, Millipore, USA). These cells can be differentiated into neurons with a dopaminergic phenotype within a short period of cultivation. Although the cells are immortalised and therefore not suitable for clinical application, the fast onset of neuronal differentiation, within 24h as shown by the change in morphology and the appearance of markers such as β-III-tubulin, make them a suitable model system for neuronal differentiation [25-28].

In our study we used the extracellular matrix protein laminin-I which promotes cell adhesion and stimulates neurite outgrowth in various neuronal cell types [34]. Cell adhesion and neurite outgrowth-promoting sites have been identified in the C-terminal site of α1 and α2 chain [35-39], in the cross-region of the molecule [40,41], in the γ1 chain [42,43], and in the N-terminal region of the α2 chain [44]. These findings support our primary choice of laminin I as a functionalisation agent in our studies.

In 2D and 3D cultures of ReNcell VM cells there are distinct variations of the growth pattern of functionalised and non-functionalised matrices. Functionalisation with laminin supports cell adhesion and prevents the formation of neurospheres. ReNcell VM cells either attach to the surface of a cell culture flask (2D) or show distinct growth patterns based on the combination of laminin with various PM scaffolds. In this context, the influence of laminin on the matrix structure is of particular interest. Structure and assembly of the functionalised matrix was investigated in detail by AFM microscopy. AFM studies demonstrate that these different growth patterns are linked to the assembly state of the 3D matrix. In general the matrix structure is built from beta-sheets and aggregates or bundles of those. Laminin directly influences the formation of the PM matrices by increasing the number of aggregates (Figure 2 and Figure 3). A PuraMatrix/laminin concentration of 0.5% has a dense and closely packed composition so that the distance between fibres is extremely small. Cells cultured in these matrices are not able to build up 3D structures, and grow in a flatter, denser pattern (Figure 5B). Decreasing the PML concentration increases the distance of nanofibres (Figure 3F, D, B) and therefore increases the possibility to form 3D growth patterns (Figure 5C). In addition, we could show that the laminin functionalisation increases the distance between fibres by shifting the composition ratio of beta-sheets to bundles more towards the bundles. Similar influence of laminin on 3D structures was reported for scaffolds consisting of Poly(l-lactic acid), whereas different modifications of the treatment of the scaffolds with laminin resulted in changes of the nanofibre structure and subsequently in the viability and proliferation of PC12 cells [45]. Regarding the changes of the scaffold into the direction of structure with more bundles, it can be concluded that this will result in 3D matrix with higher stiffness and stability. Stiffness of the surrounding matrix has important implications during development, differentiation, disease, and regeneration [46]. Although laminin clearly influences both the scaffold and the growth pattern of the cells in our study, the impact of the stiffness and stability on growth patterns and differentiation was not examined in this study and is a topic for further studies.

Besides the influence of the matrix composition on the growth and proliferation of the cells, we were interested in the question of if a 3D environment is superior to a 2D environment, as it is thought to resemble the *in vivo *situation more closely. Compared to 2D cultures the total number of cells in all matrix concentrations is significantly higher up to 7 days of differentiation, which supports the importance of a 3D environment. A question which was not addressed in the present study is the role of the non-neuronal cells. A high proportion of the cells remain positive for GFAP, suggesting differentiation into glial cells. However, this was observed in the 2D as well as the 3D culture system (data not shown).

Within this time period the survival rate of cells in the 2D culture system decreased to 70%. In contrast to our findings, Silva et al. [33] could not demonstrate significant differences in cell survival compared to 2D culture within the differentiation phase with the IKVAV hydrogel system. Hence, they suggest that diffusion of nutrients, bioactive factors, and oxygen through these highly hydrated networks is sufficient for survival of large numbers of cells for extended periods of time. The increase of cell survival in 3D matrices was also described by Mahoney and Anseth [47] with poly ethyleneglycol hydrogels. The increase in cell survival during differentiation fits very well with the higher number of β-III-tubulin positive cells, in particular after 7 days of differentiation (Figure 8). Between day 4 and day 7 of differentiation there is a drop in the number of neurons that is found in both 2D and 3D culture systems. Nevertheless, the total number of neurons remains significantly higher in 3D cultures. Another difference in the growth and differentiation potential of ReNcell VM cells is the increased number of β-III-tubulin positive neurons at day 0. Here it might be hypothesised that the functionalised matrix itself has a slightly inductive capacity towards neuronal differentiation. In addition, the growth of cells in a densely and packed environment of the matrix may induce spontaneous differentiation of progenitor cells. Furthermore, our results clearly demonstrate that the PuraMatrix/laminin 0.5% concentration failed to support the development of dopaminergic neurons at day 4 and day 7 after differentiation. In general, the number of TH-positive cells is reduced compared to 2D cultivation, except for PuraMatrix/laminin 0.25%, in which the number of TH-positive cells was higher after 7 days compared to 2D cultures. This suggests that lower concentrations support differentiation and increase the overall number of differentiated TH-positive cells. This may be due to a correlation between the structure of the matrix, namely the pores and fibres. Thonhoff et al. [23] showed that a concentration of 0.25% PuraMatrix is optimal to support stem cell differentiation, however these were not functionalised with laminin.

Proceeding from this, further studies may generate new protocols for the enrichment of neurons through manipulating the structure and composition of the matrix. Donato et al. [25] demonstrated that by pre-aggreation of the ReNcell VM cells as neurospheres before differentiation a higher number of neurons could be obtained. Another approach might be to alter the composition of the matrix with defined signalling molecules that promote neuronal differentiation. Silva et al. [33] for instance were able to demonstrate that integration of the laminin epitope IKVAV into a 3D matrix was able to initiate neuronal differentiation. Other authors [21] have demonstrated a wide variety of signalling peptides derived from laminin which have the potential to influence neurite outgrowth and differentiation. Taken together, these results show that besides the known benefit of culturing cells in 3D structures, the composition of the scaffold, specifically the concentration of the matrix material, and the functionalisation of these materials support and enhance growth and differentiation of human neural progenitor cells.

## Conclusions

In this study we demonstrated that dependent on the concentration of PuraMatrix or the functionalisation of PuraMatrix with laminin the overall structure of the 3D scaffold is changed. AFM measurements revealed changes in the 3D-structure with respect to the formation of bundles. By analysing the proliferation, differentiation and survival of human neural progenitor cells we showed that the formation and functionalisation of the 3D-structure is important for the fate of the cells. A comparison with other studies dealing with the cultivation of progenitor cells in 3D scaffolds suggests different matrix parameters may best suit different cell lines. Therefore care has to be taken regarding the matrix composition, concentration and functionalisation when using 3D systems. Taking this into account, model systems like the one we describe might be a valuable tool for the generation of defined cell types or might serve as *in vitro *assays for testing compounds which interact with the proliferation or differentiation of stem and progenitor cells.

## Abbreviations

PM: PuraMatrix; PML: PuraMatrix functionalised with laminin

## Competing interests

The authors declare that they have no competing interests.

## Authors' contributions

All authors contributed to the drafting and approval of the manuscript. SO: conception/design/analysis of immunocytochemistry data. JS: conception/design and acquisition/analysis immunocytochemistry data. SB: conception/design and acquisition/analysis of AFM data. AL: acquisition/analysis of immunocytochemistry data. LJ: conception/design and acquisition/analysis of SEM data. CAH: conception/design of AFM data.
